# Systematic Review of Socio-Emotional Values Within Organizations

**DOI:** 10.3389/fpsyg.2021.738203

**Published:** 2022-01-06

**Authors:** Tancredi Pascucci, Giuseppina Maria Cardella, Brizeida Hernández-Sánchez, Jose C. Sánchez-García

**Affiliations:** Cátedra de Emprendedores, Universidad de Salamanca, Salamanca, Spain

**Keywords:** organization, emotion, social, value, socio-emotional influence, stakeholder

## Abstract

The theory of separation assumes, with provocation, that an organization cannot reconcile profits and social function. Organizations can reconcile these two, apparently contrasting, missions, by considering emotions, especially moral emotions, to create a genuine motivation for focusing on goals beyond simple economic earnings and protecting organizations or groups of people from dysfunctional attitudes and behaviors, as well as considering the important role of the stakeholder accountability. Using the PRISMA method, we created a review of records using keywords relating to a socio-emotional value within organizations, with a particular focus on the last 20 years. We used the SCOPUS database and, after removing irrelevant records, we used the VOSviewer tool to create a cluster map of different areas in this topic. Some records cite the socio-emotional value that is related to organizational and employee suffering, while other articles consider it a positive factor that improves performance and prevents problems in organizations.

## Introduction

Entrepreneurial organizations are structures that develop ideas from all members, promoting alternative views and projects (Burger-Helmchen, 2013). Their social function aims to facilitate cohesion between people, institutions, and a collective conscience (Barbaro, 2008). For the theory of separation (Friedman, 1962; Werhane and Freeman, 1999; Alzola, 2011), social function and profits are two separate and independent missions of an entrepreneurial organization. Many authors have attempted to reconcile this dualism through research (Freeman, 2000; Freeman et al., 2004; Wempe, 2008; Hartman, 2011). The theory of separation follows an ideological dualism, whereby the entrepreneurial world (Thorpe et al., 2006), based on profit and with an individual mode, fights against a collective strategy, which aims to achieve a common good and neglects earnings. Recently, however, there has been a change due, in part, to overcome the ideological contraposition that preceded the fall of the Berlin Wall and the collapse of many state-run enterprises (Yanishev-Nesterova, 2020), when it became necessary to create a new entrepreneurial paradigm that united the entrepreneurial efficiency with a sensitivity to social, ecological, and community areas (Magala, 2002). Even before the fall of Communism, there was debate over whether an extremist approach of maximum vs. minimal state intervention was sufficient for finding a solution for the economy in some countries (Valaskakis, 1987), suggesting a strategy, in which, state institutions form partnerships with the entrepreneurial world to tackle certain problems. Social entrepreneurship is not a recent topic, having been studied since the 1970s (Moran, 1976), not only in authoritarian countries, where there is a need for the state to perform social functions to reach consensus (Tuber, 2019) but also in democratic and liberal countries, following a research path that can be considered parallel to studies about the classic entrepreneurial paradigm. In this case, there is an unavoidable need to reconcile a social mission with an earnings goal, which can be realized through rational resource management. Emotions (Graca, 2017) and moral emotions are important for giving information that could not be delivered through simple, rational, and emotionally deprived thinking, not only for an insufficient perception of reality (Miceli and Castelfranchi, 2018) but also to help us perceive the suffering and disagreement of others, to improve their lives, and to avoid anger, sadness, anxiety, and negative behavioral consequences, thereby preventing aggressive acts (Caprara et al., 2014). The quest to overcome the past division between emotions and rationality began with the ancient Greek dualism of “*pathos*” and “*logos*” and continued throughout the subsequent centuries. This contraposition was eliminated by the psychoanalytic approach (Freud, 1911; Rayner, 1995; Blanco, 2019), whereby the human mind is shown to work without following a rational line, using cognitive sciences (Beck, 1967; Arnold, 1984), in which emotion, rational processes, and cognitive functions are melted down to give us the opportunity to evaluate and manage our daily lives using the emotive intelligence paradigm (Goleman, 1996), which states that even people with the highest scores on the traditional intelligence quotient (IQ) test can perform worse than people with lower IQs but higher emotional intelligence. Emotions are important during strategic phases of organizational life, such as succession (Manzoor et al., 2018), especially in a family firm (Razzak et al., 2019). Emotions are important for reinforcing our moral and social motivations, which propel our actions toward reaching a less selfish goal that is more oriented toward equity and justice ideals. An important position in this paradigm is the stakeholder, a person or group interested in pursuing specific goals—in this case, also oriented toward social responsibility—within an organization (McVea and Freeman, 2003; Verkerk, 2013; Correa and Larringa, 2015; Barrena Martinez et al., 2016; Elizandro et al., 2018) in the areas of sport (Escamilla-Fajardo et al., 2020), education (Tolochko et al., 2020), environment (Lawrence et al., 2020), engineering (Cierna and Sujova, 2020), or medicine (Blau et al., 2012; Lierville et al., 2015; Stawicki and Firstenberg, 2018; Lomakina, 2020; Naslund, 2020).

There is a dualistic conception: An organization with a “pure” entrepreneurial philosophy, founded on profit and an individualistic view based on a zero-sum-game, where the profits of an organization are more important than the richness and wellness of society; in contrast, there is an organization that adopts a strategy strictly oriented to improving society, in terms of health, security, quality of life, or other aspects, without consideration for profit, resulting in a poor or sometimes negative economic balance (Nikodemska-Wolowik, 2008; Kirzner, 2019; Mensik, 2019). The social accounting for the stakeholder model is a paradigm, where even an entrepreneurial organization that is usually focused on satisfying only the interests of its founders and shareholders must also consider the needs of the society and the community in which it operates (Hadden, 2012; Tang and Luo, 2016; Bhatia et al., 2020; Dimitropoulos, 2020). While a traditional entrepreneurial approach involves capital investment by one person, two or more associates, or a total or prevailing state capital, there has recently appeared a new form of capital contribution, crowdfunding (Pavlidou et al., 2020). This is a solid alternative to these strategies because institutional resources are often delayed by bureaucratic procedures, while spontaneous fundraising by a private organization depends on the individual willingness of organizational associates. This strategy, which is more efficient, has been used in different areas, such as social entrepreneurship (Morell et al., 2020; Figueroa-Armijos and Berns, 2021; Motero et al., 2021), research (Dalrup et al., 2020), and start-up development (Lee and Lehdonvirta, 2020; Shi et al., 2021), to help different small investors support international entrepreneurial activity (Tiberius and Hauptmeijer, 2021) or cope with international crises, due to ecologic emergencies (Predkiewcz and Kalinowska-Beszczynska, 2020) or pandemics (Saleh et al., 2021). Previously, these areas could be treated in a sufficiently short time because, while public institutions are dedicated to managing problems that are potentially harmful to the community, they are sometimes limited by bureaucracy, political dynamics, priorities on interventions, and temporary or permanent lack of resources; meanwhile, private organizations have difficulty managing some national crises that make it necessary to switch from a profit-making to a social mindset. This difficulty emerged clearly during the coronavirus disease 2019 (COVID-19) pandemic that started in 2020 when both liberal and centralized systems faced important difficulties for the aforementioned reasons, including liberal countries with a profit-making philosophy, delayed lockdowns, and antiepidemic measures to save money and economic activities; in contrast, governments that put the wellness of people first had important difficulties for a more complex reason, that is, people in this situation were considered more important than the economy, but many workers suffered from the pause in economic activities, while many citizens also suffered a perceived loss of freedom to move due to lockdowns imposed in different countries around the world. This situation made politicians respond to different requests from people, with demands to guarantee health but also work rights, which are irreconcilable aspects; whoever has to state a priority between these two functions suffers a sort of “double bind,” risking the loss of political appreciation by choosing one over the other. Organizations must consider socio-emotional aspects without focusing only on profit-making goals and considering their social function, based on a genuine and sincere motivation, without following a consensus approach that is typically adopted by political leaders or parties, who change their priorities to maintain power (Gellatelly and Kiernan, 2003).

## Materials and Methods

We believe that socio-emotional factors are not just for consideration in clinical studies as a psychological weakness that needs to be cured but also in a positive way, as psychological wellbeing indicators or protective factors within organizational studies. Our hypotheses can be summarized as follows:

1. During the last 20 years, articles discussing the importance of socio-emotional factors have significantly increased.
2. Socio-emotional factors are not only considered negative, for example, appearing in clinical studies.
3. Socio-emotional influence is considered to be a positive factor in improving organizational management.

In this study, we considered the influence of socio-emotional factors on the life of organizations, considering both negative consequences, where a “suffering” organization is characterized by negative emotions among its members, studying its normal working and functioning considering a normal socio-emotional process, or considering records mentioning organizational strategies based on the socio-emotional aspects. We used the PRISMA statement (Liberati et al., 2009) to refine our research, using SCOPUS to obtain a literature review about this phenomenon, without considering the publication year 2021 and considering all records marked as articles or reviews, excluding conference articles, conference reviews, editorials, letters, notes, short surveys, erratum, books, and book chapters. We used a Boolean String “ORGANIZATION” AND “EMOTIONAL VALUE,” OR “SOCIAL VALUE” and excluded all pertinent records regarding non-psychological or organizational business areas. We considered only records with the following inclusion criteria:

1. Records discussing both lucrative and non-lucrative organizations.
2. Records discussing entrepreneurial organizations.
3. Records discussing organizations belonging to public institutions.
4. Records written in English.

We also stated exclusion criteria and did not consider records defined by the below keywords within their subject area because they did not involve a psychological aspect:

• Biochemistry
• Medicine
• Mathematics
• Earth Sciences
• Physics

Once we refined a list of records on SCOPUS, we used VOSviewer (Waltman et al., 2010) to create a cluster analysis using author keywords and excluding redundant keywords, reusing SCOPUS to cite them within each cluster.

## Results

We extrapolated 1,761 records. The first mention citing the influence of socio-emotional value on organizations appears in a publication of 1922 (Link, 1922), describing a Psychological Service Centre, where, despite strong stress on individual psychology among first clinical psychological contributions, there is a significant sensibility about organizational functioning, going beyond simple individual psychology. After this first work, there were no further articles for almost 20 years, with some articles after the 1960s about therapeutic groups and health organizations. If we consider the number of records during each decade between the 1960s and the 2020s, as we can observe in Table 1, we notice an exponential incrementation of studies about this area, with more than 50 articles published by the 1990s. We hypothesized that the importance of socio-emotional influence on organizations is due to the birth of interest in emotions (Burton, 1963; Krebs, 1975; Hoppe, 1983; Goodnow, 1990) outside clinical and experimental psychology, which, up to the second half of the twentieth century, were exclusively treated within psychotherapy studies and laboratory walls.

**TABLE 1:** Number of records for each decade with more of 50 records publication and incrementation publishing rate.

Publishing years	Records No.	% Incrementation rate
1991–2000	103	110%
2001–2010	403	289%
2011–2020	1201	202%

**FIGURE 1:**
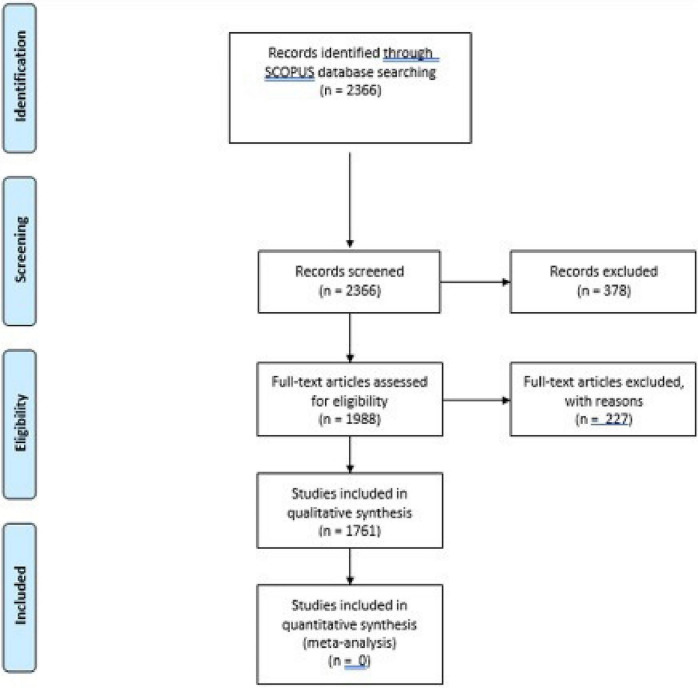
Graphical representation of records number during last 20 years.

Continuing to represent a graphical trend in Figure 1, we revealed that this trend has grown over the last 20 years, with a vail between 2005 and 2010 (probably due to the important economic crisis during 2007–2009) and a good incrementation after 2015.

**FIGURE 2:**
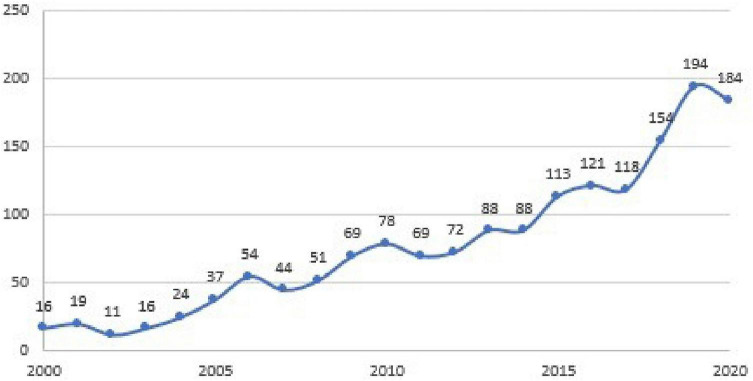
Graphical representation of most active publishing countries.

If we consider countries that have published at least 30 records about this topic, we can observe in Figure 2 that the United States is the highest publishing country, with a significant gap to others. The United States, the United Kingdom, and Australia are the first three countries treating this topic, showing the supremacy of an Anglo-Saxon research approach, even though many Asian and European countries contribute.

Considering most active journals as presented in Tables 2, 3, we underline how much socio-emotional value is a core topic in most business management publications, where the organization has to consider the variables in order to have a peculiar sensitivity to satisfying community requests and to preserve a positive organizational climate and individual wellness among personnel. Some journals in this area have an h-index superior to 40, showing how much this area attracts important research teams.

**TABLE 2:** List of most active journals about this research line.

No.	Journals	h-index	Research area
32	Journal of Management Development	55	Business and Management Accounting; Organizational Behavior and Human Resource Management
30	Journal of Organizational Change Management	66	Business, Management Accounting; Decision Sciences
29	Journal of Managerial Psychology	74	Business, Management Accounting; Decision Sciences, Psychology
29	Personnel Review	67	Business Management Accounting; Psychology
26	Leadership and Organization Development Journal	62	Business, Management and Accounting; Organizational behavior and Human resource management
18	Journal of Services Marketing	96	Business, management and accounting
16	Employee relations	48	Business, management and accounting; Organizational Behavior and Resource Management
15	Human Resource Management International Digest	11	Organizational Behavior and Resource Management
15	International Journal of Conflict Management	50	Business, management and accounting; Social Sciences
15	Management Decision	91	Business, management and accounting; Decision Science

**TABLE 3:** List of authors with more of 4 publications about socio emotional influence.

No.	Author	Affiliation	h-index	Prevailing research area
5	Applebaun, S., H.	John Molson School of Business, Montreal, Canada	27	Business Management Accounting; Social Sciences; Decision Sciences.
4	Boyatzis, R., E.	Cleveland University, United States	31	Business Management Accounting; Psychology; Social Sciences; Economics, Econometrics.
4	Dolan, S., L.	ESADE, Barcelona, Spain	12	Business Management Accounting; Psychology; Social Sciences; Medicine; Economics, Econometrics.
4	Eisenberger, R.	University of Houston, United States	40	Psychology; Social Sciences; Business Management Accounting; Medicine.
4	Karatepe, O., M.	Mersin University, Turkey	44	Business Management Accounting; Social Sciences; Economics, Econometrics and Finance; Environmental Sciences
4	Mesler, G.	Haifa University, Israel	6	Business Management Accounting; Psychology; Economy, Econometrics; Social Sciences; Medicine.
4	Srivastava, S.	Jaipur Institute of Management, Noida, India	4	Business Management Accounting; Social Sciences; Economy, Econometrics; Decision Sciences; Psychology.

Despite the Anglo-Saxon supremacy of the literature, if we consider the most productive authors, there are many contributions from Spanish, Turkish, Israeli, and Indian researchers. The most prolific author on this list is Applebaun from a Business School in Montreal, who has written articles about leadership and stereotypes among organizations (Applebaun et al., 2016c). Emotional intelligence is an important area of interest for various authors (Boyatzis, 2009; Boyatzis and Ratti, 2009; Gabel-Shemueli and Dolan, 2011; Meisler, 2014): Eisenberger, affiliated with the Psychology Department in Houston, focuses on organizational perceived support, another important aspect in the wellbeing of employees (Ameli et al., 1998; Kim et al., 2016; Kurtessis et al., 2017), and Karatepe and Srivastava are Turkish and Indian researchers, respectively, who publish articles about negative socio-emotional aspects regarding organizations, such as burnout or work-family conflict (Karatepe and Tekinus, 2006; Karatepe and Zargar-Tizabi, 2011; Karatepe, 2015; Srivastava et al., 2019; Srivastava and Argawal, 2020; Srivastava and Dey, 2020).

**FIGURE 3:**
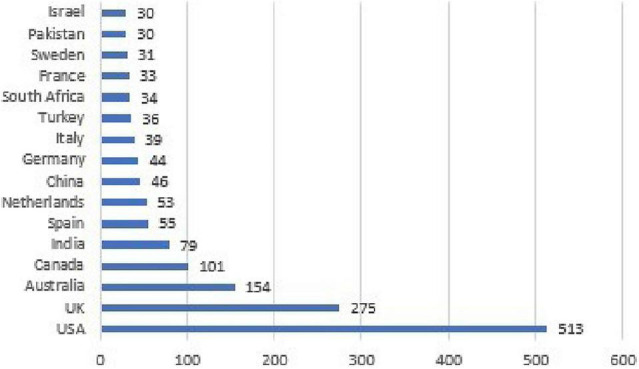
Graphical representation of cluster map author’s keywords.

We used VOSviewer to divide the previous group of articles on SCOPUS, using their author keyword co-occurrence. We stated a minimum of 10 occurrences, extrapolating 53 items. We revealed a graphical representation, which was regrouped into six different clusters. These clusters, with their co-occurrence, are graphically represented in another image in Figure 3.

## Cluster (Red Colored) 1: Emotional Exhaustion (15 Items, 318 Records)

This is the cluster regrouping with the highest record number (18% prevalence) and recalls significant distress within organizations (Peng et al., 2010; Reed et al., 2018), regarding both users and personnel, where the psychological suffering of the first damages the second and vice versa. Stress (Deckard and Present, 1989; Micael et al., 2008; Richardsson et al., 2008; Martinèz et al., 2013; Sheldon et al., 2015; Linden et al., 2018; Prada-Ospina, 2019), anxiety (Goetz et al., 2006; MacNeil et al., 2009; Inal et al., 2010; Shongwe and Cilliers, 2020; Wu et al., 2020), and depression (LeDoux et al., 1998; Galvin et al., 2006; Jafri et al., 2011; Karatepe and Zargar-Tizabi, 2011; Rooney and Grant, 2013; Wu et al., 2020) are principal indicators of this psychological symptomatology, sometimes reaching dangerous levels and becoming a psychiatric emergency (Johnson et al., 2016; Gil-Rivas et al., 2019). This vulnerability makes personnel members uncomfortable, causing burnout (Booth and Faulkner, 1986; Gupchup et al., 1994; Robinson et al., 2003; Skaalvik and Skaalvik, 2011; Rama-Maceiras et al., 2012; Glaser and Hect, 2013; Huynh et al., 2014; Malik et al., 2016), which has important consequences not only at the individual level but also to family and working performance (Glaser and Hect, 2013; Babic et al., 2020).

### Cluster (Green Colored) 2: Emotional Intelligence (12 Items, 297 Records)

This cluster is the second most numerous cluster (17% prevalence) regarding socio-emotional aspects (Crusheil, 2006; Kerr et al., 2006; Akerjordet and Severinsson, 2007; Hopkins et al., 2007; Koman and Wolff, 2008; Coskun et al., 2018; Chen et al., 2019; Perez-Fuentes et al., 2019), which can create a buffer effect against distress within organizations, preventing individual and organizational suffering. Organizations are not purely rational institutions (Carter, 2005) where every individual has a precise role, with tasks to perform and intervention protocols to manage automatically with plenty of clear satisfaction and without ambiguities and inner conflicts (Chiva and Alegre, 2008; Meisler, 2014). People who work are not always satisfied with their personal and working life and clearly change jobs constantly. They are sometimes frustrated by difficulties, and working skills cannot be evaluated, excluding emotional components. Emotional intelligence (Chrusciel, 2006; Suliman and Al-Shaikh, 2007; Davies et al., 2010), since its introduction by Goleman (1996), defines an important ability to cope with problems that cannot be navigated using only intellectual skills (Brown et al., 2006), considering also moral aspects that a pure rational intelligence does not consider, thereby creating learning opportunities (McCracken, 2005; Vorhauser-Smith, 2011; Starbuck, 2017). Emotional intelligence can be used within organizations to prevent emotional exhaustion (Meisler, 2014; Clark and Polesello, 2017), to manage and create an appropriate organizational climate (Der Foo et al., 2004; Morehouse, 2007; Chen et al., 2016) that improves organizational performance (Gabel-Shemueli and Dolan, 2011; Wei and Li, 2011; Bettis-Outland and Guillory, 2018) and leadership (Kent, 2006; Fowlie and Wood, 2009; Hess and Bacigalupo, 2010; Lindebaum and Cartwright, 2011; Applebaun et al., 2016a,b, c, d) and provides organizations with an ethical approach (Rok, 2009; Fairchild, 2010; Capell and Gabell-Shemueli, 2013; Caldwell and Hayes, 2016; Asgary and Lawrence, 2020).

### Cluster (Blue Colored) 3: Organizational Culture and Knowledge Sharing (8 Items, 89 Records)

An important aspect of organizations is the cooperation between different workers within their organization (Shihi and Susanto, 2010). This aspect, apparently simple and reasonable, is sometimes forbidden, bringing the workers of an organization to work without coordination (Cangemi et al., 2008; Daniels, 2009; Kahili, 2017). This means that different departments or workers from the same organization could work on the same task doing intervention already carried out by the other (Meisler and Vigoda Gadot, 2014), or sometimes performing counterproductive actions (Zhang and Shi, 2017), just like a psychiatrist who prescribes psychotropic drugs with significant collateral effects for a cardiopathic patient cured by another physician. Organizations have their peculiar organizational climate (Armeli et al., 1998; Worthington, 2012), their myths (Gordon and Winpenny, 1996; Bao et al., 2013), their mission and objective (Campbell and Yeung, 1991; Rahmani et al., 2015; Krajcsàk, 2018; Serwint and Stewart, 2019), group resilience, and power (Freeman and Carson, 2006; Leggat and Balding, 2013; Seyedpour et al., 2020), where the individuality of every single worker should be directed at the same goal (Flanagan and Henry, 1994; Leggat and Balding, 2013; Gokturk et al., 2017; Herkes et al., 2019). Better cooperation and communication are important for trust between different workers (Dibben, 2004; Swift and Hwang, 2013; Lim et al., 2018), which grants better cooperation and knowledge sharing, preventing useless or chaotic actions.

### Cluster (Yellow Colored) 4: Care Services Communications (7 Items, 175 Records)

Some organizations and professions have a superior emotional investment (Penticuff, 1989; Ho, 2007; Hujala and Rissanen, 2011) than those in which the professional has to stress less about the rational and technical aspects of their activities. A classic example is the helping professions, where helpers have scientific protocol interventions to use but cannot exclusively manage their job without considering socio-emotional aspects (Ramo et al., 2009; Fairchild, 2010). Nurses (Severinsson and Hallberg, 1996; Tuck and Wallace, 2000; Wright and Baker, 2005; Evans, 2006; Stone et al., 2006; Strandas et al., 2019) are typically one of the principal professions dedicated to superior socio-emotional nearness to patients (Brunton, 2005; Waddington and Fletcher, 2005; Ostaszkiewicz et al., 2016). If we compare their relationship with a more detached physician-patient relationship (Dubler, 1995), they are the principal person who constantly manages the illness of a patient, even more than a parent or other sanitarian workers. Considering that, socio-emotional influence is not only fundamental to preventing a psychological collapse in these cases but also to improving caregiver-patient relations and communication (Thomson and Hecker, 2001; Loosemore, 2010; Vinall-Collier et al., 2016), with a positive effect on the healing process (Rustoen, 1998; Molter, 2003). This interaction is a result of two personalities of the patient-nurse dyad, comprehending both normal and sometimes pathological but functional personalities (Dijkstra et al., 2005; Young and Dulewitz, 2007; Igarashi et al., 2009; Wolff and Kim, 2012; Lounsbury et al., 2016; Schwarz et al., 2016).

### Cluster (Violet Colored) 5: Gender Issue (6 Items, 250 Records)

Female workers are considered more oriented to emotions than men; it is the third most representative cluster (i.e., 14% prevalence), and female workers are typically involved in some working activities, such as nursing (Severinsson and Hallberg, 1996; Tuck and Wallace, 2000; Wright and Baker, 2005; Evans, 2006; Stone et al., 2006; Gray, 2009; Strandas et al., 2019) and school education (Salami, 2008; Galtseva et al., 2020). Some of these activities are more complicated than others and sometimes less socially considered (Syed et al., 2005; Gray, 2009, 2010; Blau et al., 2012; Yagil, 2014; Hur et al., 2015; Shin et al., 2015; Pandley, 2018; Adams and Mastracci, 2020; Dhliwayo and Coetze, 2020). This cluster focuses on both positive and negative emotions (Donnay et al., 1993; Wilkinson, 2002; Humphreys et al., 2005; Driller et al., 2011; Mularz and Johansen, 2016).

### Cluster (Light Blue Colored) 6: Hospital and Quality of Life (5 Items, 207 Records)

This cluster is located within the walls of the hospital and is treated by intervention protocols based on the socio-emotional influence (Berman et al., 2016). Hospital patients cannot be cured using only medication and pharmacological therapies and must be considered as human beings with their own personalities and desires, needs, and fears. Medicine must consider life wellness and satisfaction in patients, especially for medical pathology, where psycho-social aspects and quality of life of patients (Powell and Kornfeld, 1993; Knight et al., 2001; Erim et al., 2015) contribute to a better disease progression, considering the severity of their clinical condition, which can be chronic, mild, or permanent (Duncan and Siegal, 1998; Sengelov et al., 2000; Tsunoda et al., 2007; Segalla et al., 2008; Inal et al., 2010; Yu et al., 2010). Palliative therapies in this case, sometimes have significant prognostic effectiveness despite usually being used in terminal-illness departments, and the decision-making process (Solloway et al., 2005; Oliver and Jacobs, 2007; Gronstad, 2017; Bakst et al., 2019) during therapy is a fundamental aspect in diagnosing and acting at the right moment during the healing process.

## Discussion

**FIGURE 4:**
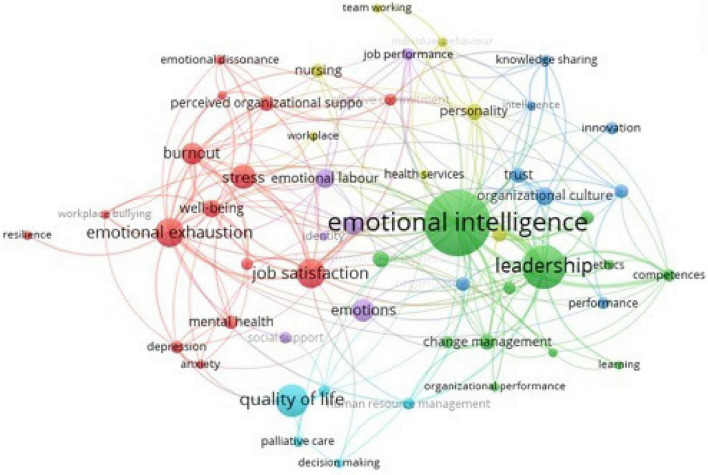
PRISMA statement about socioemotional values in organizations.

This study did not only consider emotional (Lombard, 2021) or social (Micaelson, 2021) values because these constructs are often used interchangeably, even if there are some important differences. In contrast to the “hard” sciences, we treated a research area full of disturbing variables as decision science, where we must adopt a complexity paradigm (Jorm et al., 2021). Considering a negative attitude through socio-emotional influence, people who use an emotional approach are irrational and potentially target manipulation (Lenidou and Lenidou, 2009; Yang, 2016). Our topic analysis uses cluster mapping with two orthogonal Cartesian axes in Figure 4, giving a graphical setting, where the horizontal defines a cluster mostly centered on individual wellness or, in contrast, defining an organizational setting, while the vertical is more oriented to a negative consequence/state or a positive way.

In a synthetic description, the position of every record under the same cluster could be in a different graphical area. For example, burnout is a keyword under cluster 1. Many authors define burnout as a syndrome characterized by individual psycho-physic suffering, which can also regard some causes and consequences related to the entire organization of a worker, defining this construct and its cluster negatively, but connected at both an individual and organizational level (Schulz et al., 1995; Yip and Rowlinson, 2009; Brown and Quick, 2013; Jones-Schenk, 2019). It emerges as a balanced result, whereby cluster 1, with the highest number of co-occurrence, is related to psychopathological problems, but the other clusters are positively related to socio-emotional values, where there is a positive influence that improves protective aspects in organizations. The emotional intelligence or other aspects bring potential improvement to organizational performance in terms of a more efficient organization and communication (clusters 3 and 4), personal skills, and inner positive characteristics for better interpersonal competence (clusters 2 and 5) or considering the psychological needs of users and patients to improve their wellness (cluster 6).

Our three hypotheses have been verified by this review.

1. If we consider the last 20 years of research, we notice an incrementation in records considering both emotions and organizational keywords, following a general incrementation trend, whereby emotions are not neglected considering their influence among organizations. There were mild decreases during times of economic recession, for example, during the economic crises of 2008 and 2020, with the beginning of the economic recession caused by movement limitations due to COVID-19, following a new increase.
2. Our cluster analysis revealed prevalence and where emotions are considered negatively, with discussions of stress, burnout, depression, and clinical manifestations due to emotional dysfunction, but we underline that it is just a cluster, whereas the others talk about leadership, gender issues, and organizational performance due to an ethical approach, which also considers emotions as “humanizing” to an organization.
3. Emotions also relate to organizational management, whereby emotions cannot be considered only in an individual way, as single human characteristics, but are also related to organization and people groupings.

For this research, we found many records, even considering only English-language articles and reviews and only using the SCOPUS database to focus on the research topic. This must be underlined as a limitation, as we excluded other databases.

## Conclusion

People working within an organization and the same management rules are strongly moved by a purely rational mechanism, and problems that organizations treat cannot be solved following only rational logic. Kahneman and Tversky (1981), Kahneman (2003), and Thaler and Sunstein (2008), demonstrate that emotions strongly determine important human decisions, even significant financial investments. Recalling the beginning of this study, the theory of separation brings an organizational attitude that spoils the organization from their social function, creating an extremist world consideration, in which an organization cannot consider the possibility of earning money by considering the common good, neglecting environmental sensitivity, labor and political rights, urban sustainable development, and creating wars and exploitation. Trying to overwhelm an ideological approach, the integration of profit and social function must involve socio-emotional elements among organizations introducing social accountability (Hadden, 2012; Tang and Luo, 2016; Bhatia et al., 2020; Dimitropoulos, 2020), which aims to obtain a balanced0 vision between these two dimensions. Emotions are fundamental to moving managers to a major social sensitivity and an ethical approach to business management (Rok, 2009; Fairchild, 2010; Capell and Gabell-Shemueli, 2013; Caldwell and Hayes, 2016; Asgary and Lawrence, 2020), better communication (Dibben, 2004; Swift and Hwang, 2013; Lim et al., 2018), and improved organizational performance (Gabel-Shemueli and Dolan, 2011; Wei and Li, 2011; Bettis-Outland and Guillory, 2018), preventing conflict (Flanagan and Henry, 1994; Leggat and Balding, 2013; Gokturk et al., 2017; Herkes et al., 2019) and suffering (Micael et al., 2008; Richardsson et al., 2008; Skaalvik and Skaalvik, 2011; Martinèz et al., 2013; Rooney and Grant, 2013; Shongwe and Cilliers, 2020). There has been an important incrementation rate during the last 20 years on this topic probably due to the generalized growth of the research. The economic crisis of 2008–2009 reopened discussion of the importance of social function among entrepreneurial organizations, where the exaggerated free economic speculation of single businessmen damaged Western economies; it was the failure of a liberal system that equaled the fall of the Berlin Wall almost 20 years before, where, on the contrary, there was a socialistic failure with an overcentralized economy, which limited entrepreneurial freedom, creating a closed and not competitive market. The COVID-19 pandemic revealed significant neglect of the socio-emotional influence of human behaviors.

Many countries around the world have delayed a tempestive and preventive intervention and have underestimated the pandemic problem in favor of a short-term economic decision strategy, which does not consider long-term effects and consequences (Zang et al., 2020). Different enterprises, such as fashion firms (Zara, Armani, Zegna, H&M, Gucci, etc.), have reconverted some of their factories for the production of medical instruments. Surely, the smart working, already existing, will receive a better diffusion and application, and any service or selling activities are improving in the educational, medical, or selling sectors (Soled et al., 2020; Spurlock, 2020). An important intervention in many countries is the educational system, changing the teaching style, no longer being based on face-to-face relations (Choe and Choi, 2020). There is now a confrontation between economic superpowers, and Europe has to participate as a single country formed by different nations. There is some financial European aid, which is implementing different economic aid programs for each individual European nation. Moreover, there are—beyond the usual European projects that the EU promotes annually—different specific European projects, such as Pan European Hackaton #EUvsVirus, which regroups different people from every European country, with different preparations who create different projects to fight the emergency using different focus areas.

This strategy is predominantly insufficient and prioritized economic and sanitarian aspects, where the latter mostly considered just medical aspects without considering a bio-psycho-social model (Engel, 1977), which could properly set a better preventive intervention strategy, avoiding a delay of contention strategy against the virus and with chaotic management of the health emergency, where a late intervention by health workers only partially coped with the biological risk, but almost totally neglected the psycho-social negative consequences of the pandemic, creating important discomfort among the population (Griffith, 2020; Banerjee et al., 2021; Jokic-Begic et al., 2021; Szmulewitz et al., 2021; Taylor et al., 2021). COVID-19 is not only a pandemic, which has caused the death of millions of people, but it has also seriously damaged world economies, creating a psycho-social impairment among the population and acting negatively toward organizational vulnerabilities among work and health organizational systems, considering, in this case, only the economic priority, with biological risk as a secondary consideration. However, even in this case, only the medical aspect of this drama was considered, neglecting plenty of effective prevention (better than a cure!) being realized by considering psychological intervention (Hanna-Attisha and Olson, 2021; Mimiaga et al., 2021; Zhang et al., 2021). It is difficult to maintain critical thinking in this case, and often people fail in this task because it is easier to follow emotional thinking (Wynes, 2021; Yu et al., 2021), which has often negatively influenced socio-political decisions, for example, creating and maintaining populist movements, which are stronger in uncertain times (Bone, 2021). Stakeholder accountability moves its actions through a complex situation, especially during an actual socio-economic crisis, where there is a significant impairment in terms of social cohesion and trust between people and institutions (Antinyan et al., 2021; Fikukova et al., 2021; Surina et al., 2021; Ye et al., 2021). The accountability of stakeholders means that a single person or a group of people operate for an individual initiative, without an institutional setting, following their own principles, intrinsic rules, and ethics (Salsbury et al., 2018). There are some easy ways to lead in this situation, in which, people must cope with another great challenge: to cooperate with the institution and other people within the same organization or become an autocratic leader, taking every decision alone, deciding easier and faster, but forgetting a democratic approach and risking important mistakes (Zarinah et al., 2017; Hentschel et al., 2018; Levene and Higgs, 2018). History has many examples of “gifted” people in moral and leadership terms who take charge, with all the decision-making power in their hands and guide the destiny of entire countries.

## Data Availability Statement

The original contributions presented in the study are included in the article/supplementary material, further inquiries can be directed to the corresponding author/s.

## Author Contributions

TP and GC stated keywords and build the cluster analysis. TP contributed to write and sampling statistics. BH-S and GC contributed to discussion. All authors contributed to the article and approved the submitted version.

## Conflict of Interest

The authors declare that the research was conducted in the absence of any commercial or financial relationships that could be construed as a potential conflict of interest.

## Publisher’s Note

All claims expressed in this article are solely those of the authors and do not necessarily represent those of their affiliated organizations, or those of the publisher, the editors and the reviewers. Any product that may be evaluated in this article, or claim that may be made by its manufacturer, is not guaranteed or endorsed by the publisher.
